# Conformational landscapes resolved by ion mobility mass spectrometry reveal mechanisms of polyubiquitin-controlled phase separation

**DOI:** 10.1039/d6sc00836d

**Published:** 2026-05-19

**Authors:** Christina G. Robb, Thuy P. Dao, Jakub Ujma, Carlos A. Castañeda, Rebecca Beveridge

**Affiliations:** a Department of Pure and Applied Chemistry, University of Strathclyde Glasgow G1 1XL UK rebecca.beveridge@strath.ac.uk; b Departments of Biology and Chemistry, BioInspired Institute, Syracuse University Syracuse New York 13244 USA; c Waters Corporation Stamford Avenue, Altrincham Road Wilmslow SK9 4AX UK

## Abstract

Polyubiquitin chains regulate phase separation of the ubiquitin-binding shuttle protein ubiquilin-2 (UBQLN2) in a manner that depends on the position of the isopeptide linkage and the length of the polyubiquitin chain. However, conformational heterogeneity of the non-covalent complexes formed between these proteins has rendered the molecular mechanisms underlying this regulation elusive. Here, we have used ion mobility mass spectrometry (IM-MS) to disentangle conformational features of the non-covalent complexes formed by UBQLN2 with different polyubiquitin chains. We demonstrate that the length of the polyubiquitin chain binding to UBQLN2 has a large effect on the conformational distribution of the complex, with increasing chain lengths (up to four ubiquitin subunits) allowing access to more extended conformations of the complex, and shorter chains promoting compaction. This length-dependent modulation of conformational landscapes provides the evidence for distinct mechanisms of phase separation regulation encoded by ubiquitin chain length. We also compare polyubiquitin chains linked by lysine residue (K)48 and by K63, both alone and in complex with UBQLN2. By understanding how K48- and K63-linked tetraubiquitin chains behave in the gas phase, which differs to solution, we propose molecular mechanisms for their regulation of UBQLN2 phase separation. This work, enabled by the unique ability of IM-MS to resolve conformational features of highly heterogeneous biomolecular systems, represents a conceptual breakthrough in understanding polyubiquitin-controlled phase separation mechanisms.

## Abstract 2

This work outlines conformational features of highly dynamic complexes formed between different polyubiquitin chains and ubiquilin-2, and represents a conceptual breakthrough in understanding polyubiquitin-encoded phase separation mechanisms.

## Graphical abstract

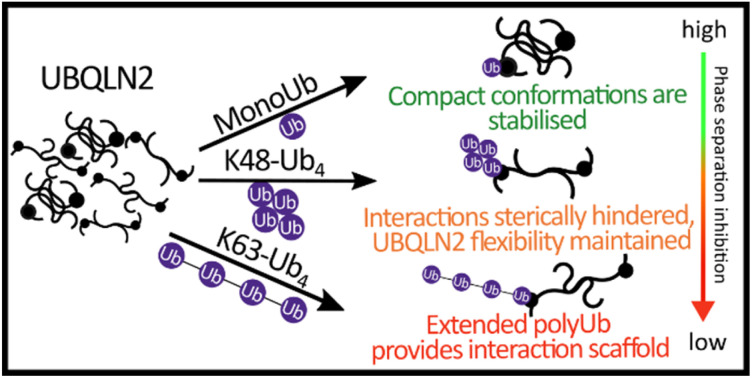

## Introduction

Across all cell types, a balance between protein synthesis and degradation must be strictly maintained for proper cellular function, in a process known as proteostasis. In eukaryotic cells, many protein quality control (PQC) mechanisms ensure proper protein folding by preventing aggregation and removing damaged or misfolded proteins.^1^ Defects in PQC mechanisms are associated with disease states including cancer and neurodegenerative diseases, often due to a build-up of misfolded protein deposits.^2^ Two major ways that proteins are recognised and disposed of are *via* the ubiquitin-proteasome system (UPS) and the autophagy-lysosome pathway.^3,4^ In the first step of both processes, proteins undergo covalent attachment of ubiquitin (Ub), which acts as a marker for proteins to be degraded.^5,6^ Ub can be attached to the target protein either as a single protein or as a polyubiquitin (polyUb) chain.^7^ The isopeptide linkage in polyUb chains is located at seven lysine (K) side chains, with K48 and K63 being the most prominent sites, as well as the *N*-terminal methionine (M1).^5,8^ The location of the linkage within the Ub chain partly determines the fate of the attached substrate within the cell. K48-linked chains typically signal for proteasomal degradation, a process involving the 26S proteasome machinery, which disposes of small and soluble proteins.^9^ K63-linked chains are implicated in autophagy which, in contrast, disposes of large protein aggregates or more long-lived proteins in the cell.^10,11^ However, this network is complex, as there are also situations where substrates labelled with K63-polyUb are degraded by the proteasome, especially *in vitro*. Additionally, branched polyUb chains containing both K63 and K48 linkages are recognised by the proteasome.^12,13^

**Fig. 1:**
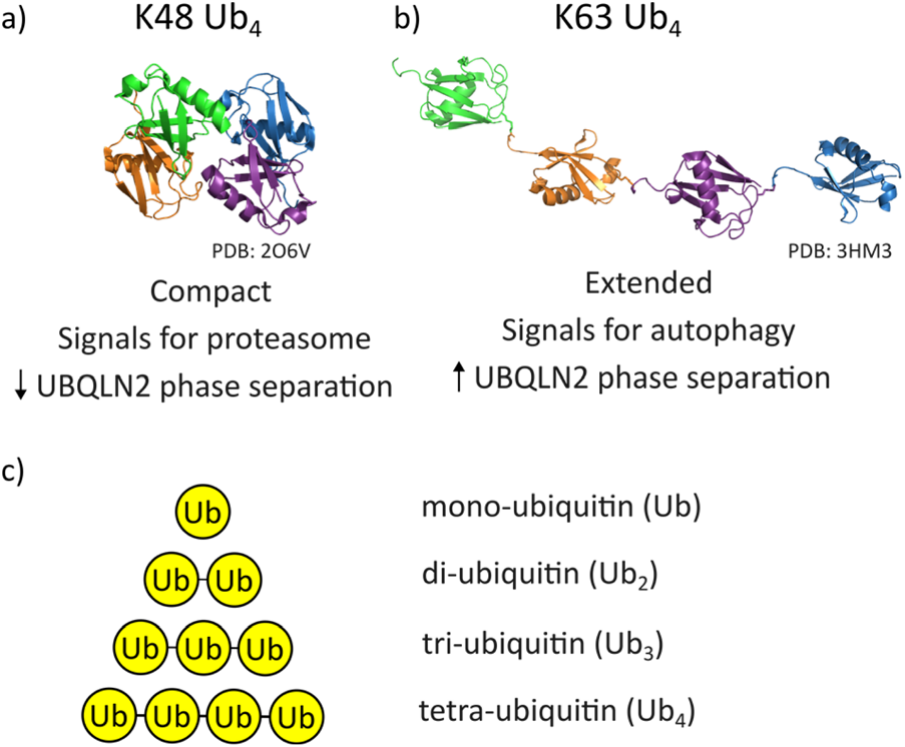
(a) Representative K48-Ub_4_ structure (PDB file 2O6V). It has a compact conformation and signals for proteasomal degradation in cells. When in solution with UBQLN2, it disrupts UBQLN2 phase separation in a concentration-dependent manner. (b) K63-Ub_4_ structure (PDB file 3HM3). It has an extended conformation and signals for autophagy in cells. When in solution at sub-stoichiometric concentrations with UBQLN2, it promotes UBQLN2 phase separation. (c) Schematic of polyubiquitin constructs used in this study: Ub, di-ubiquitin (Ub_2_), tri-ubiquitin (Ub_3_) and tetraubiquitin (Ub_4_). In all cases, each Ub subunit is linked by an isopeptide linkage between the two molecules, where the linkage is either K48 or K63.

Besides signalling for different pathways, differentially-linked polyUb chains exist in different conformations. K48-linked tetraubiquitin (K48-Ub_4_) adopts a globular-like, compact conformation in which the hydrophobic patches of the individual Ub molecules may interact (Fig. 1a).^14^ K63-linked tetraubiquitin (K63-Ub_4_), on the other hand, has a beads-on-a-string extended conformation (Fig. 1b).^14,15^ Despite good understanding of these different conformations, the mechanisms of how different polyUb chains signal for different degradation pathways remain currently limited.^16^

Ub-binding shuttle proteins, such as ubiquilin-2 (UBQLN2), interface between ubiquitinated substrate proteins and their degradation pathway.^16^ UBQLN2 consists of a ubiquitin-like domain (UBL)^17^ and a ubiquitin-associating domain (UBA) that interact with the protein degradation machinery and Ub-linked substrates, respectively. An integral process to the function of some shuttle proteins is phase separation, which results in the formation of membraneless compartments (biomolecular condensates) within the cell.^18^ Phase separation is commonly enabled by multivalent interactions encoded within folded and disordered domains of proteins.^19–21^ UBQLN2 is recruited to stress granules, a type of membraneless organelle that forms in response to cellular stresses such as heat shock.^22^ Ubiquitination is also upregulated during stress, and polyUb chains affect the dynamics of stress granule formation and disassembly.^23^ The effect of Ub and differentially-linked polyUb chains on phase separation of UBQLN2 has been studied *in vitro*, yielding interesting results.^24,25^ Ub is a potent inhibitor of UBQLN2 phase separation, even at sub-stoichiometric concentrations. K48-Ub_4_ also disrupts the process, but requires higher concentrations than Ub. In contrast, K63-Ub_4_ promotes UBQLN2 phase separation when it is present at low concentrations, and then has an inhibitory effect only when a large excess is present.^24^ By transitioning between dilute and condensed phases, Ub-binding shuttle proteins can modulate the fates of ubiquitinated substrates.^24^

Another key feature of Ub-binding shuttle proteins, as well as many other proteins that can undergo phase separation, is that they contain high proportions of intrinsic disorder. Intrinsically disordered proteins (IDPs) or proteins containing regions of intrinsic disorder (IDRs) are characterised by their ability to fluctuate over a wide range of conformations rather than possessing a well-defined, stable structure.^26^ This dynamic nature of IDPs makes them perfectly suited to be involved in protein–protein interaction networks, such as the protein degradation pathways.^27^ Despite their importance in cell signalling networks and their high representation in disease states, IDPs are challenging to study as their lack of stable structure hinders the use of classical structural biology techniques such as X-ray crystallography for determining structural information.^28^ More recently, native mass spectrometry (nMS) and ion-mobility mass spectrometry (IM-MS) have been demonstrated to be applicable to the study of IDPs.^29,30^

We previously used IM-MS to investigate the conformational dynamics of UBQLN2 alone and in complex with Ub.^31^ IM-MS is an effective method for studying IDPs and complexes involving IDPs as it reveals the full range of conformations in which they exist. Intact proteins and protein complexes are maintained during nanoelectrospray ionisation (nESI), enabling stoichiometric information to be gained regarding the complexes formed. From this, we demonstrated that UBQLN2 exists as a mixture of monomers and dimers, and that the dimeric form can bind either 1 or 2 Ub molecules, resulting in 2 : 1 and 2 : 2 UBQLN2 to Ub stoichiometries. Structural information can also be inferred from the number of charges carried by the protein or protein complex, as compact conformations will have a smaller surface area to accommodate protons and will have a low charge state, whereas extended conformations will have the ability to carry more protons and will have a higher charge state. A wide charge state range is a hallmark of IDPs, and represents the ability of the protein to exist in a wide range of conformations.^32,33^ From this information, we identified that UBQLN2 dimers are highly disordered, whereas the complexes formed with Ub had a significantly smaller conformational range.^31^ IM-MS provides further insights into the conformations, as different species are separated according to their overall size. This allows multiple conformations to be identified from a single charge state.

Here, we have expanded the use of IM-MS to study the complexes formed between UBQLN2 and differentially-linked Ub chains, ranging in length from 1–4 Ub subunits. First, from analysing the Ub_4_ chains alone, we identified that K63-Ub_4_ undergoes compaction in the gas phase to a geometry that is smaller than K48-Ub_4_. We then explored the relationship between polyUb length and its impact on the conformational range of UBQLN2, demonstrating that complexes involving Ub chains of increasing length are increasingly dynamic. Finally, we compared the conformations of UBQLN2 : Ub_4_ involving K48-Ub_4_ and K63-Ub_4_, and identified that K63-Ub_4_ undergoes a similar compaction event as its unbound form. Taken together, these measurements have allowed us to propose distinct mechanisms for how different polyUb chains modulate UBQLN2 phase separation behaviour.

## Methods

### Protein expression, and purification

Wildtype, K48R, K63R, Ub and Ub-V-His6 were expressed and purified as detailed elsewhere.^34,35^ The gene encoding mouse E1 was a kind gift from Jorge Eduardo Azevedo (Addgene plasmid 32 534,^36^). E1, Mms2, Yuh1 and GST-Ubc13 were expressed in *Escherichia coli* NiCo21 (DE3) cells in Luria–Bertani (LB) broth at 16 °C overnight. GST-E2-25K in pGEX-4T2 was expressed in *Escherichia coli* Rosetta 2 (DE3) pLysS cells in Luria–Bertani (LB) broth at 16 °C overnight. Bacteria were pelleted, frozen, then lysed *via* freeze/thaw method in 50 mM Tris, 1 mM EDTA (pH 8), 1 mM PMSF, 1 mM MgCl_2_, and 25 U of Pierce universal nuclease. Yuh1, E1 and Mms2 were purified *via* Ni^2+^ affinity chromatography. GST-E2-25K and GST-Ubc13 were purified *via* GST chromatography. All proteins were concentrated, buffer exchanged into 50 mM Tris and 1 mM EDTA (pH 8) and stored at −80 °C for subsequent use in the production of K48-Ub_4_ and K63-Ub_4_.

K48-linked and K63-linked Ub_2_, Ub_3_ and Ub_4_ were synthesized sequentially. Briefly, equal amounts of K48R (K63R) Ub and Ub-V-His6 incubated with 1000 nM E1 and 10 µM GST-E2-25K (2 µM His-Mms2 and 4 µM GST-Ubc13) in the presence of 10 mM ATP, 0.3 mM TCEP in Tris buffer at pH 8 for 3 hours at 37 °C. This procedure generates K48R (K63R) Ub2 with the *C*-terminal end of the proximal Ub blocked by V-His6. Yuh1 was added to remove the V-His6 from the end of Ub2, which was then purified *via* cation exchange column using 50 mM ammonium acetate (AmAc, pH 4.5) as the buffer. Protein was eluted *via* a linear gradient from 0 to 100% of 50 mM AmAc, 1 M NaCl (pH 4.5). Purified Ub_2_ was then buffer exchanged into 50 mM Tris buffer at pH 8. K48- and K63-Ub_3_ were made the same way Ub_2_ was made. K48 and K63-Ub_4_ required an additional purification step *via* size exclusion chromatography using a Superdex 75 HiLoad 16/600 column (GE Healthcare). The yield for K48- and K63-Ub_4_ was about 50%.

Full-length UBQLN2 was expressed and purified as described previously.^37^ Briefly, the construct was expressed in *E. coli* Rosetta 2 (DE3) pLysS cells in LB broth at 37 °C overnight. Bacteria were pelleted, frozen, lysed, then purified *via* a “salting out” process. NaCl was added to the cleared lysate to the final concentration of 0.5 M–1 M. UBQLN2 droplets were pelleted and then resuspended in 20 mM NaPhosphate, 0.5 mM EDTA, 0.1 mM TCEP, 0.02% NaN_3_ (pH 6.8). Leftover NaCl was removed through HiTrap desalting column (GE Healthcare). Purified proteins were frozen at −80 °C.

### Preparation of proteins for nMS and IM-MS

UBQLN2 (30 µM) was buffer exchanged into 10 mM AmAc pH 6.8 using 96-well microdialysis plates (Thermo Fisher Scientific, Waltham, MA USA). Ub, Ub_2_, Ub_3_ and Ub_4_ were buffer exchanged using Bio-Rad Micro Bio-Spin P6 columns (Bio-Rad, Hercules, CA, USA). Final protein concentrations were determined using a Implen NP80 nanophotometer (Implen, München, Germany) using the A280 method. Protein concentrations were calculated with extinction coefficients of 11 460 M^−1^ cm^−1^ for UBQLN2, 1490 M^−1^ cm^−1^ for Ub, 2560 M^−1^ cm^−1^ for Ub_2_, 3840 M^−1^ cm^−1^ for Ub_3_ and 5960 M^−1^ cm^−1^ for Ub_4_.

### nMS and IM-MS measurements

UBQLN2 was diluted to a protein concentration of 15 µM and final AmAc concentration of 10 mM (pH 6.8) for experiments on the unbound protein. For experiments with UBQLN2 and Ub_2_, Ub_3_ and Ub_4_, a 1 : 1 molar ratio of UBQLN2 monomer to Ub was mixed by adding 12 µM of UBQLN2 (10 mM AmAc) to an equal volume of 12 µM of Ub_2_, Ub_3_ or Ub_4_ (10 mM AmAc). Samples were allowed to equilibrate on ice for at least 30 minutes. For experiments on Ub_2_, Ub_3_ and Ub_4_ alone, samples were diluted to a protein concentration of 5 µM in 100 mM AmAc.

IM-MS data were acquired on a Waters Synapt G2-Si (Waters Corporation, Wilmslow, UK) instrument with an 8 k quadrupole operated in a “Sensitivity” mode. Proteins were subject to nESI in positive mode with a nanoelectrospray emitter pulled in-house with a Flaming/Brown P-97 micropipette puller from thin-walled glass capillaries (i.d. 0.78 mm, o.d. 1.0 mm, 10 cm length, both from Sutter Instrument Co., Novato, CA, USA). A positive potential of 0.9–1.4 kV was applied to the solution *via* a thin platinum wire. Other non-default instrument settings are sampling cone voltage 60 V, collision voltage 5 V, trap gas flow 3.5–4 mL min^−1^, and source temperature 40 °C. IM data of UBQLN2 alone was collected at traveling-wave velocity of 400 m s^−1^ and height of 40 V. IM data of UBQLN2 complexes with Ub_3_ and Ub_4_ were collected at traveling-wave velocity of 325 m s^−1^ and ramped height of 25–40 V. IM data of Ub_2_ alone was collected at traveling-wave velocity of 225 m s^−1^ and ramped height of 22.5–37.5 V. IM data of Ub_3_ alone was collected at traveling-wave velocity of 225 m s^−1^ and ramped height of 25–40 V. IM data of Ub_4_ alone was collected at traveling-wave velocity of 200 m s^−1^ and ramped height of 25–40 V. IM parameters were optimised for individual protein constructs. Helium and nitrogen (IMS) gas flows were 180 and 90 mL min^−1^. The instrument was allowed to settle for 1 h prior to experiments. A manual quadrupole RF profile of *m*/*z* 3750 was applied to improve the transmission of ions from *m*/*z* 2750 and upward in ion mobility mode.

### Data processing

Mass spectra were initially processed in MassLynx v4.2 (Waters Corporation, Wilmslow, UK). IM-MS profiles were created in OriginPro 2022 (OriginLab Corporation, Northampton, MA, USA) by extracting arrival time distributions (ATDs) of selected charge states in the mass spectrum. For UBQLN2 : Ub_4_ complexes, normalised data from multiple days (*n* = 3 or 4) was averaged and the standard deviation was calculated. For Ub_2_, Ub_3_, Ub_4_ and UBQLN2 : Ub_3_ complexes, normalised data from three measurements across two days were averaged and standard deviation was calculated. Standard deviation was calculated in both instances using the Descriptive Statistics function in OriginPro 2022 and reported as error, shown by the shaded region on the ATDs.

A Welch's *T*-test was performed to compare the intensity at the indicated points (*y* = 6.359) in Fig. 4a and b using Hypothesis Testing (Two Item *T*-Test) in OriginPro 2023 (*n* = 3, *M* = 0.65171, SD = 0.154, DF = 2.49297, *T* (2) = 3.58, *p* = 0.05).

## Results

### K63-Ub_4_ is more compact than K48-Ub_4_ in the gas phase

**Fig. 2:**
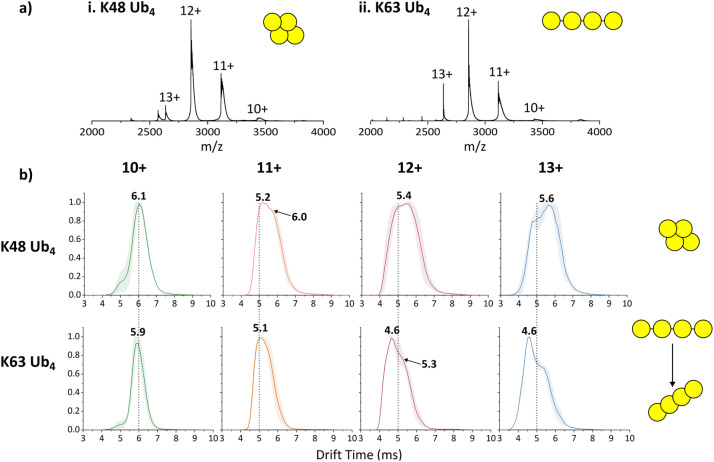
nMS (a) and IM-MS (b) of K48- and K63- Ub_4_ analysed at an initial concentration of 5 µM from 100 mM AmAc. The number in bold above each peak in (b) is the peak apex. Dotted lines are to guide the eye for comparison between tetraubiquitin types.

K48-Ub_4_ and K63-Ub_4_ were first analysed by nMS as an initial conformational assessment (Fig. 2a). Each Ub_4_ is present in 4 charge states, ranging from 10+ to 13+, with 12+ being highest intensity and both 10+ and 13+ observed at relatively low intensity. The charge state distribution (CSD) is almost identical in each spectrum, suggesting compact conformations for both species.^32^

Next, we used IM-MS to measure the arrival time distributions (ATDs) of each charge state of K48-Ub_4_ and K63-Ub_4_ (Fig. 2b), to further interrogate any differences in the conformational distributions of the two constructs. ATDs can also be converted to rotationally averaged collision cross section (CCS) values, reported in nm^2^, through the use of calibration strategies. However, the identical molecular composition of the two Ub_4_ chains, and their equivalent charge states, allow direct comparison of ATDs and preclude the necessity of any calibrations. The ATDs of the 10+ charge state for both species are relatively narrow and have a similar arrival time (6.1 and 5.9 ms for K48-Ub_4_ and K63-Ub_4_ respectively), suggesting similar conformations for this lowest charge state. The ATDs corresponding to the 11+ charge state have a very similar apex, but for K48-Ub_4_ there is also a partially-resolved peak at 6.0 ms corresponding to a larger conformation, which is not observed in the ATD of K63-Ub_4._ This unexpected observation, in that K48-Ub_4_ is larger than K63-Ub_4_, is also reflected in charge states 12+ and 13+. The ATD corresponding to 12 + K48-Ub_4_ is very broad, with the existence of two conformations being indicated by a primary apex at 5.4 ms and a secondary apex around 5.0 ms. The peak shape for 12 + K63-Ub_4_ is very different; there is a sharp apex at 4.6 ms, which is 0.8 ms shorter than that of K48-Ub_4_, followed by a shoulder on the right-hand side of the peak (5.3 ms). The 13+ charge states follow a similar trend to the ATD for the 12+ species, with the apex drift time being 1.0 ms shorter for K63-Ub_4_ than K48-Ub_4_. Overall, as charge increases, the ATD corresponding to K63-Ub_4_ is consistently narrower and shorter than that of K48-Ub_4_, indicating that the former has a more compact conformation in the gas phase, which is in contrast with well-documented solution-phase behaviour. These relatively broad IM-MS profiles, containing partially resolved features, are typical of protein analytes due to their inherent dynamicity, even for globular examples.^38,39^

We rationalised our results by examining the protein structures obtained by X-ray diffraction, NMR and SAXS (Fig. 1).^14,15^ K48-Ub_4_ is a globular species, where all four ubiquitin subunits interact with each other *via* hydrophobic patches. Their subunits are physically close to each other stemming from the geometric constraints imposed by the K48 isopeptide bond. Conversely, K63-Ub_4_ is a more linear species, with gaps between each subunit where the K63 isopeptide linkage between two Ub subunits resides. We hypothesise that during desolvation, the space between the covalently linked ubiquitin molecules is lost, resulting in a highly compact conformation of K63-Ub_4_ in the gas phase.

To investigate the generality of this gas phase compaction of K63-Ub_4_, we also compared ion mobility profiles of K63- and K48-linked Ub_2_ and Ub_3_ (Fig. S1 and S2). In agreement with the data pertaining to Ub_4_, in both cases, the K63-linked Ub chains are more compact than those linked *via* K48.

### The length of the polyUb chain strongly affects the dynamic propensity of the resulting UBQLN2 : Ub_*n*_ complex

**Fig. 3:**
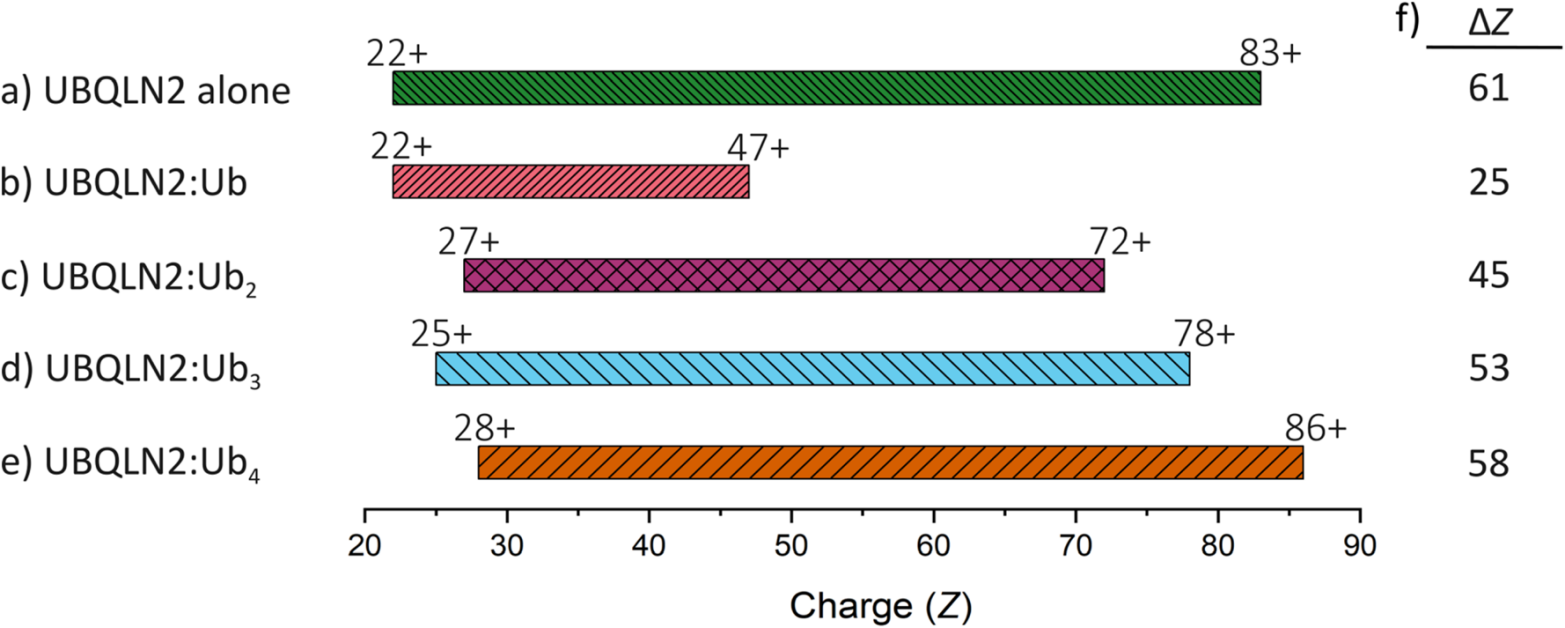
CSDs plotted as a bar graph of dimeric UBQLN2 alone (a) and bound to monoUb (b), Ub_2_ (c), Ub_3_ (d) and Ub_4_ (e). In the case of Ub_2_, Ub_3_ and Ub_4_, the results are identical when the isopeptide linkage is *via* K63 or K48. Labels above the beginning and end of each bar represent lowest and highest charge detected for each species. (f) charge range or Δ*Z* for each complex. As the number of ubiquitin units in the chain increases, the Δ*Z* also increases.

We next compared the dynamic behaviour of complexes formed between UBQLN2 and differential polyUb chains containing 1–4 Ub subunits (Fig. 3). nMS is a useful method for this comparison, as the resultant charge state range is reflective of the range of protein conformations, with lower charge states corresponding to compact conformations and higher charge state corresponding to extended geometries.^32^ In the absence of Ub, UBQLN2 dimers display a charge state distribution of 22+ to 83+ (Fig. 3a and S3), with a charge state range (Δ*Z*) of 61, which corresponds to a highly dynamic protein that exists in a wide range of conformations.^31^ Upon binding one molecule of monoUb to form a 2 : 1 UBQLN2 : monoUb complex (Fig. 3b and S4), the lowest charge state remains the same as the unbound UBQLN2 (22+) but the highest charge state is reduced from 83+ (unbound UBQLN2) to 47+ (monoUb-bound UBQLN2), resulting in a reduced Δ*Z* of 25. This vast reduction in Δ*Z* reflects lower dynamicity of the UBQLN2 : monoUb complex compared to the unbound form. We explored this previously and proposed that UBQLN2 compaction by monoUb binding is an underlying mechanism for inhibition of UBQLN2 phase separation,^31^ as there are fewer exposed sites available for forming the weak multivalent interactions required for the process. Complexes in a stoichiometric ratio of 2 : 2 UBQLN2 : monoUb were also identified, which have a similar charge state distribution to the 2 : 1 complex (24+ to 49+). However, as these 2 : 2 species were only detected to a very low extent that prevented robust data interpretation, only the 2 : 1 complexes were considered in this study.

Upon increasing the number of Ub subunits in the chain to two (Ub_2_, Fig. 3c, S5 and S6), the charge state range is 27+ to 72+, with Δ*Z* increasing dramatically to 45 (*c.f.* 25 for the monoUb complex). This shows that the complex formed with Ub_2_ exists in a wider range of conformations compared to that with monoUb, and suggests that Ub_2_ stabilises compact conformations of UBQLN2 to a lower extent. This trend persists with longer chains. UBQLN2 : Ub_3_ complexes (Fig. 3d, S7 and S8) range from 25+ to 78+ (Δ*Z* = 53), and UBQLN2 : Ub_4_ complexes (Fig. 3e, S9 and S10) range from 28+ to 86+ (Δ*Z* = 58). While we cannot draw firm conclusions about the lowest charge states due to detection limitations in this *m*/*z* range, there is a clear trend in the highest charge states: as the number of Ub subunits in the covalently linked chain increases, the maximum charge state and Δ*Z* also increase. This allows us to hypothesise that the longer the polyUb chain, the lesser extent to which compact conformations of UBQLN2 are stabilised and this allows UBQLN2 to retain more flexibility. It also allows us to tentatively suggest that monoUb is causing compaction by rearranging the conformational ensemble of UBQLN2. A partial explanation may stem from disruption of intramolecular UBL : UBA interactions that could rebalance other interactions throughout the protein including the IDRs (see Discussion). Experiments were performed using both K48- and K63-Ub_*n*_ species, but no differences in the charge states of the complexes were observed based on the isopeptide linkage (SI Fig. S5–S10).

### IM-MS measurements of Ub_4_ : UBQLN2 complexes reveal how differentially-linked polyUb molecules regulate UBQLN2 phase separation

As previously mentioned, the mass spectra corresponding to the UBQLN2 : Ub_4_ complexes were extremely similar for the K63-linked and K48-linked polyUb chains. We therefore sought to use IM-MS to further interrogate conformational differences between these complexes that have been previously identified using NMR, fluorescence anisotropy and analytical centrifugation (AUC).^25^ It has been suggested that UBQLN2 complexes involving K63-Ub_4_ are more extended than those involving K48-Ub_4_, but these results were limited in that the technical requirements for these methods prevented the use of full-length UBQLN2. One hypothesis was that K63-Ub_4_ stabilises a more extended conformation of UBQLN2. Another was that K63-Ub_4_ acts as an ‘extension’ to UBQLN2 in solution, providing a greater interaction network that promotes UBQLN2 phase separation.

**Fig. 4:**
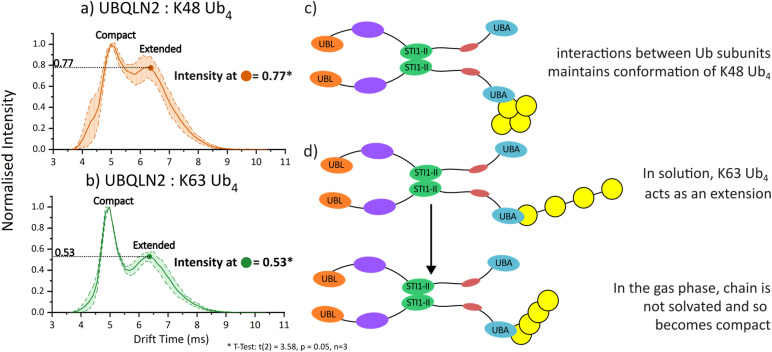
Arrival time distributions of (a) 2 : 1 UBQLN2 : K48-Ub_4,_ (b) 2 : 1 UBQLN2 : K63-Ub_4_ 41+ charge state. The solid line represents average of three measurements across two days and the shaded area within dotted lines represents the standard deviation across measurements. The normalised intensity of the longer drift time species is 0.77 for K48-Ub_4_ complex and 0.53 for K63-Ub_4_ complex, which is indicated by a filled circle on each graph. Cartoon representations of (c) UBQLN2 : K48-Ub_4_ and (d) UBQLN2 : K63-Ub_4_ interaction. *T*-test (see asterisk) confirms the different in intensity is statistically significant (*p* = 0.05), see methods for more details.

The ATD of the 41+ charge state of the UBQLN2 : K48-Ub_4_ consists of two distinct peaks with apexes at 5.0 ms and 6.4 ms, which correspond to a compact conformation and a more extended conformation, respectively (Fig. 4a). The ATD of the UBQLN2 : K63-Ub_4_ also has two peaks with the same arrival times as UBQLN2 : K48-Ub_4_, but that differ in their relative intensities (Fig. 4b). The later-arriving peak, corresponding to the more extended conformation, has a relative intensity of 0.8 for K48-Ub_4_, but only 0.5 for K63-Ub_4_. These results suggest a larger population of the more extended conformation for UBQLN2 : K48-Ub_4_ compared to UBQLN2 : K63-Ub_4_, which is in disagreement with previous solution-phase results but follows on from our (and others') observations for the Ub_4_ species alone.^40^

Thus, we propose that UBQLN2 exhibits the same or very similar dynamic properties when bound to either type of Ub_4_ chain, which in turn is very similar to the unbound form of UBQLN2 based on the charge state distributions (Fig. 3). We therefore suggest that the difference between the complexes in the IM-MS measurements is due to the behaviour of the Ub_4_, and that these differences reflect the behaviour of Ub_4_ in isolation, *i.e.* that K63-Ub_4_ undergoes compaction in the gas phase, whereas the K48-linked version remains robust. Again, in these measurements, the identical molecular composition of these complexes allowed direct comparison of arrival time measurements without calibration to CCS values.

Our interpretation of these results is that K63-Ub_4_ acts as an extension of UBQLN2 in solution and is only anchored by the interaction between a single Ub unit and the UBQLN2-UBA. Upon desolvation of the intact complex, the K63-Ub_4_ lacks any stabilising interactions with the rest of the UBQLN2 molecule that would enable it to remain extended, and hence becomes compacted in the gas phase (Fig. 4d). K48-Ub_4_ on the other hand, with its globular conformation, is more stable and is therefore resistant to gas-phase compaction, in a similar manner to its unbound form (Fig. 2 and 4c). The logic regarding our proposal of this solution-phase mechanism based on gas-phase measurements is consolidated in a flow chart (Fig. S11). The UBQLN2 : K63-Ub_3_ complex also shows a more compact conformation compared to UBQLN2 : K48-Ub_3_ complex (Fig. S12).

## Discussion

### Conformations of free polyubiquitin chains

The first aim of this study was to characterise the conformational flexibility of K48- and K63-Ub_4_ using IM-MS, with the initial hypothesis that the former would preferentially exist in a more compact conformation than the latter. This hypothesis was based on the wealth of data pertaining to the behaviour of these constructs in the solution phase using techniques such as NMR, SAXS and AUC.^41^ In MS, the CSDs displayed by these proteins are almost identical. However, IM-MS revealed that each charge state of K63-Ub_4_ is more compact than K48-Ub_4_. This trend is also observed for both Ub_2_ and Ub_3_ polyUb chains. To rationalise these results, we suggest that during desolvation of K63-Ub_4_ the space between the covalently-linked Ub molecules is lost, resulting in a highly compact conformation of K63-Ub_4_ in the gas phase. K48-Ub_4_, on the other hand, is already a more globular species and retains its conformation during desolvation. These results regarding the unbound form of K48- and K63-Ub_4_ are supported in a recent study by Jung *et al.*^40^ in which these proteins were analysed *via* IM-MS from denaturing solution conditions. It was reported that K63-Ub_4_ is larger than K48-Ub_4_ only at the highest charge states (34+ to 37+) but at lower charge states (22+ to 33+), K63-Ub_4_ is smaller than K48-Ub_4_ which agrees with our measurements from native-like conditions.

Our results regarding the gas-phase compaction of K63-linked chains are also important to consider alongside additional biomolecules containing flexible linker regions.^42^ Significant compaction is not observed for antibodies IgG1, IgG2 and IgG4 which contain short flexible linker regions, nor for dinucleosomes, which are two nucleosome core particles, linked together by a flexible region of DNA.^43–45^ However, a high degree of compaction is observed for IgG3, which interestingly contains a longer flexible hinge region than the other antibodies.^45^ From these observations, we hypothesise that molecules containing mid-length flexible linkers do not infer enough disorder for the protein to undergo the chain ejection mechanism (CEM) of electrospray ionisation,^46^ or the recently described bead ejection mechanism.^47^ Instead, they will undergo the charged residue mechanism similar to globular proteins,^48^ but will undergo a high level of compaction which is allowed by the flexibility of the interdomain linker.^49^ Better understanding and ability to predict this phenomenon will be enabled by further research combining experimental and theoretical approaches,^50,51^ and for now, IM-MS results corresponding to molecules with a predicted ‘beads-on-a-string’ architecture should be considered alongside orthogonal, solution phase methods, as we have performed in this study.

### Effect of chain length on complex dynamics

**Fig. 5:**
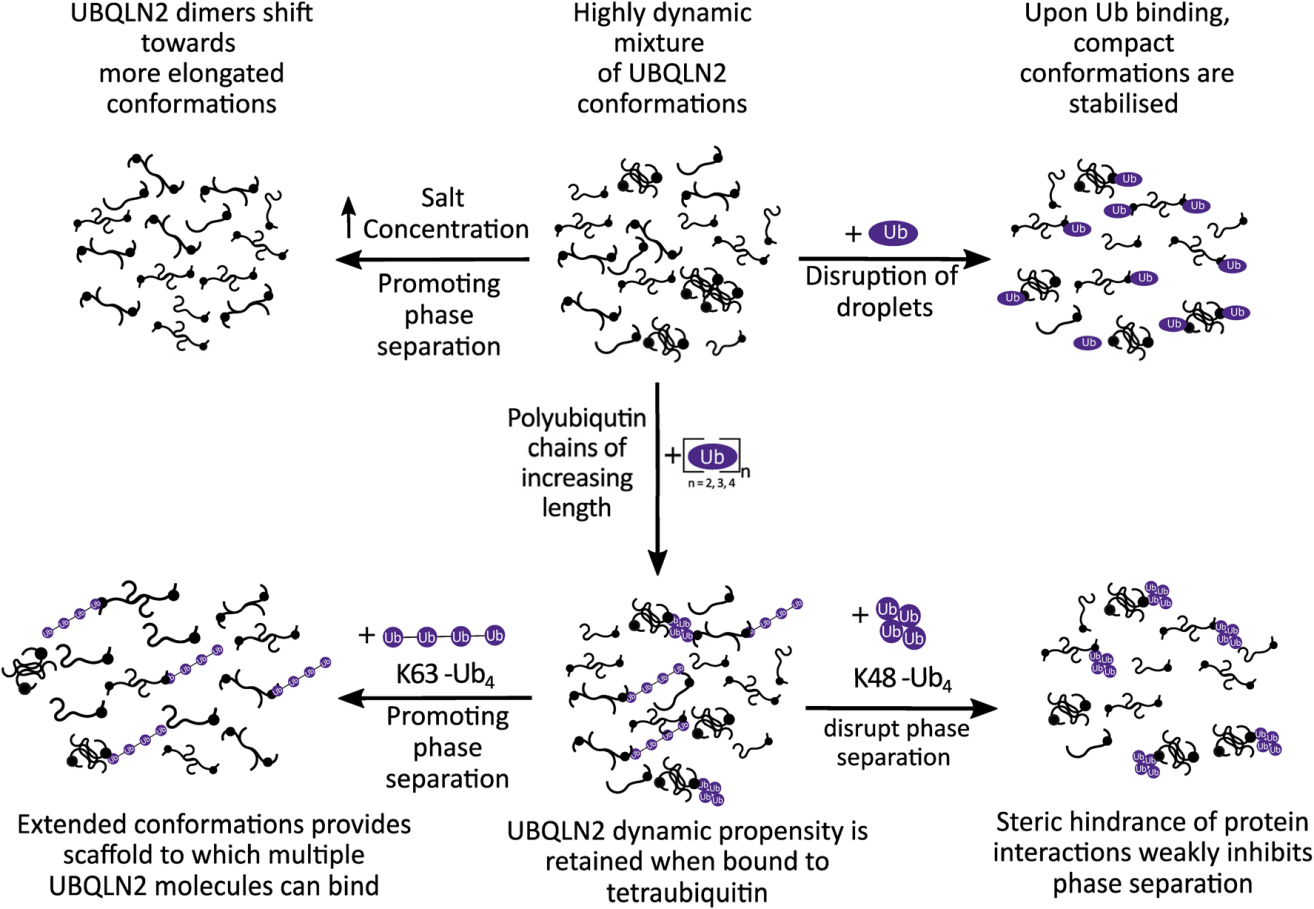
Model for how UBQLN2 behaviour is modulated by interaction with different ubiquitin species, and how this may impact phase separation. MonoUb is a potent inhibitor and promotes stabilisation of compact conformations. Ub_4_ does not cause the same compaction as monoUb, instead allowing UBQLN2 to retain most of its dynamic propensity in its bound form. We hypothesise that K48-Ub_4_ weakly inhibits by steric hindrance, such that multiple UBQLN2 molecules are unable to bind to a single K48-Ub_4_. In contrast, we propose that K63-Ub_4_ promotes phase separation by providing extended scaffolds upon which multiple UBQLN2 molecules could bind, enhancing multivalent interactions among the UBQLN2 molecules.

In previous work we identified that monoUb stabilises compact conformations of UBQLN2, and here we sought to investigate whether this was true for Ub_*n*_ chains of increasing length.^31^ We identified that the CSD of UBQLN2 : Ub_*n*_ complexes becomes broader as Ub_*n*_ increases from 1–4 Ub subunits. This suggests that binding of longer chains of Ub_*n*_ to UBQLN2 stabilise compact conformations of UBQLN2 to a lesser extent. This length-dependent modulation of conformational landscapes provides evidence that longer Ub chains inhibit UBQLN2 phase separation by different mechanisms compared to monoUb (Fig. 5).

We can also infer information regarding the specific regions of UBQLN2 that are compacted upon the binding of monoUb and shorter Ub chains. Whilst it is widely accepted that Ub binds to the UBA of UBQLN2, we tentatively suggest that monoUb is causing compaction by disrupting multivalent interactions across UBQLN2, such as known UBL : UBA interactions and consequently other interactions between these domains and the IDRs of UBQLN2.^52^ This rebalancing of interactions could result in the highest loss of protein flexibility and hence the biggest decrease in CSD. By considering that the compaction is correlated with a decrease in phase separation propensity, we can further suggest that these multivalent interactions are being formed with the PXX domain, which confers the temperature sensitivity to phase transitions,^53^ and with the STI1-II domain, which promotes dimerisation and further oligomerisation.^22^ These assertions would benefit from further research using cross-linking-MS to localise interaction interfaces.

It is interesting to note the lack of linkage dependence on the CSDs of UBQLN2 : Ub complexes containing di- tri- or tetra-Ub. As described above, this is likely due to the flexible linker regions in K63-linked Ub chains being too short to enable ionisation of these constructs *via* the CEM. Therefore, differences in the phase separation propensity of K48- and K63-linked Ub chains are more likely to be related to conformational differences inferred from IM-MS data, as described below.

### Ub chain-specific mechanisms of phase separation regulation

Our IM-MS results allow us to consider the effect of different Ub and polyUb constructs on the propensity of UBQLN2 to phase separate. Previous research has shown that monoUb is the strongest inhibitor of UBQLN2 phase separation, and that polyUb chains of increasing length display a weaker inhibitory effect. MonoUb causes a significant compaction of UBQLN2, as shown by the narrower CSD of UBQLN2 : Ub compared to UBQLN2 alone, whereas the UBQLN2 in UBQLN2 : Ub_4_ complexes remains highly dynamic. Our results therefore provide evidence that monoUb and polyUb inhibit UBQLN2 phase separation *via* different mechanisms. Since monoUb causes UBQLN2 compaction whereas Ub_4_ does not, we suggest that the latter may use its conformation to prevent multiple UBQLN2 molecules from interacting with the same polyUb chain and thus decreasing the propensity for phase separation to occur. Ub_4_ may also act as a steric hindrance by preventing the UBQLN2 chains from coming into close enough proximity (Fig. 5). This model is primarily based on our results pertaining to K48-Ub_4_. Whilst K63-Ub_4_ behaves similarly in terms of CSD, subtle conformational differences reflected in the IM-MS data and their potential impact are described below.

Our results regarding K48-Ub_4_ relate to previous research in which NMR spectroscopy was used to show that its binding to a UBQLN2 construct consisting of residues 450–624 affects regions outside the UBA domain (residues 580–620). Specifically, K48-Ub_4_ affects residues 555–570, a putative helical region in the intrinsically disordered linker of UBQLN2.^25^ Since the binding of K48-Ub_4_ reduces phase separation propensity of UBQLN2, it is tempting to speculate that its binding occludes interaction interfaces such as in the STI1-II region, but this is yet to be confirmed *via* experimental data. On the other hand, monoUb binding to UBQLN2 causes overall compaction of UBQLN2, thereby reducing the available surface area of UBQLN2 and reducing the propensity for weak multivalent interactions required for phase separation.

K63-Ub_4_, however, causes an increased propensity for phase separation at low concentrations, followed by inhibition at high excess of Ub_4_.^25^ We propose that K63-Ub_4_, in either the UBQLN2-bound or unbound state, is dynamic in solution and then compacts as it enters the gas phase. During phase separation, we speculate that this dynamic extension to UBQLN2 acts as a scaffold and potentially permits additional UBQLN2 molecules to bind to other Ub units in the K63-Ub_4_ chain (Fig. 5). In turn, this additional UBQLN2 binding could increase the localised UBQLN2 concentration, hence promoting UBQLN2 : UBQLN2 interactions and further promoting phase separation. This assertion is supported by analytical ultracentrifugation (AUC) data reported by Dao *et al.*,^25^ which indicate that multiple copies of a *C*-terminal UBQLN2 construct (residues 487–624) bind more readily to a single K63-Ub_4_ molecule than to K48-Ub_4_. We attribute this to the extended conformation of K63-Ub_4_ which increases accessibility of Ub-binding interfaces relative to the more compact K48-Ub_4_. Whilst no higher-order stoichiometries beyond 2 : 1 are observed in our MS experiments, this could be further interrogated by charge-detection mass spectrometry (CD-MS) which is better suited for larger protein complexes.^54^ This current working model relies on inferences from the data that are currently indirect, and would benefit from further interrogation using solution-phase measurements such as crosslinking-MS or smFRET.^55,56^

Overall, our ability to propose phase separation mechanisms comes from the conformational information that can be obtained for mass-selected species in an nMS experiment, using full-length UBQLN2. Such analysis is a valuable advancement to prior work using NMR and AUC with shorter UBQLN2 constructs, as these methods face limitations with the complexity incurred during use of the full-length protein. As there is no bias towards complexes of specific sizes, we can detect that K48-Ub_4_ does not reduce the solvent-accessible surface area, thus the charge state range, of UBQLN2 upon complexation and so doesn't result in the same compaction event we observed for Ub.

It has been proposed that K63-linked ubiquitinated substrates are protected from degradation by remaining inside the condensate whereas K48-linked substrates are not protected as phase separation is disrupted and thus, are degraded by the proteasome.^24^ This work supports this hypothesis by providing insight into distinct conformations resulting from UBQLN2 interacting with K48- and K63-Ub_4_. The existence of such distinct conformations for K48- and K63- Ub_4_ alone and in complex with UBQLN2 may aid in explaining how UBQLN2 is capable of shuttling ubiquitinated substrates to the proteasome, *via* K48 linkages, and to autophagy and other non-proteolytic pathways, *via* K63 linkages.

## Conclusion

This work provides a new working model for the mechanistic effect of polyUb chain architecture, in terms of its length and linkage type, on the phase separation propensity of UBQLN2. Through the use of IM-MS and its unique ability to delineate conformational landscapes within heterogeneous mixtures, we have elucidated a new working model which links binding complex architecture, conformational features, and phase separation mechanisms. Our results, along with published data from solution-phase techniques,^24,25^ provide a new perspective to how the ubiquitin code is engineered to direct ubiquitinated substrates to different functional outcomes *via* different polyubiquitin linkages through conformational control.

## Author contributions

R. B. and C. A. C. conceptualised the study. R. B. and C. G. R. designed the experiments. C. G. R. performed experiments, and analysed the mass spectrometry and ion-mobility data with input from R. B. and J. U. C. A. C. and T. P. D. expressed and purified UBQLN2, Ub, Ub_2_, Ub_3_ and Ub_4_. C. G. R. prepared the initial draft of the manuscript with input from R. B. All authors contributed to manuscript review, editing, and discussion.

## Conflicts of interest

The authors declare the following competing financial interest(s): J.U. is an employee of Waters Corporation (Wilmslow, UK) who manufactures and sells IM-MS instrumentation.

## Supplementary Material

SC-017-D6SC00836D-s001

## Data Availability

Raw mass spectra and ion mobility spectra corresponding to Fig. 2–4 are available in both.RAW and.xlsx formats at Strathclyde Pure Portal [https://doi.org/10.15129/256d0168-5581-4ed8-b84d-311d453b614d]. Additional data supporting the findings of this work are included in the supplementary information (SI). Supplementary information: Fig. S1–S12. See DOI: https://doi.org/10.1039/d6sc00836d.

## References

[cit1] McClellan A. J., Tam S., Kaganovich D., Frydman J. (2005). Protein quality control: chaperones culling corrupt conformations. Nat. Cell Biol..

[cit2] Webster C. P., Smith E. F., Shaw P. J., De Vos K. J. (2017). Protein Homeostasis in Amyotrophic Lateral Sclerosis: Therapeutic Opportunities?. Front. Mol. Neurosci..

[cit3] Grice G. L., Nathan J. A. (2016). The recognition of ubiquitinated proteins by the proteasome. Cell. Mol. Life Sci..

[cit4] Ohsumi Y., Mizushima N. (2004). Two ubiquitin-like conjugation systems essential for autophagy. Semin. Cell Dev. Biol..

[cit5] Komander D., Rape M. (2012). The Ubiquitin Code. Annu. Rev. Biochem..

[cit6] Weissman A. M., Shabek N., Ciechanover A. (2011). The predator becomes the prey: regulating the ubiquitin system by ubiquitylation and degradation. Nat. Rev. Mol. Cell Biol..

[cit7] Swatek K. N., Komander D. (2016). Ubiquitin modifications. Cell Research.

[cit8] Chen Z. J., Sun L. J. (2009). Nonproteolytic functions of ubiquitin in cell signaling. Mol. Cell.

[cit9] Liu Z. (2019). *et al.*, Structural basis for the recognition of K48-linked Ub chain by proteasomal receptor Rpn13. Cell Discov..

[cit10] Pickart C. M., Fushman D. (2004). Polyubiquitin chains: polymeric protein signals. Curr. Opin. Chem. Biol..

[cit11] Glickman M. H., Ciechanover A. (2002). The Ubiquitin-Proteasome Proteolytic Pathway: Destruction for the Sake of Construction. Physiol. Rev..

[cit12] Ohtake F., Tsuchiya H., Saeki Y., Tanaka K. (2018). K63 ubiquitylation triggers proteasomal degradation by seeding branched ubiquitin chains. Proc. Natl. Acad. Sci. U. S. A.

[cit13] Nathan J. A., Tae Kim H., Ting L., Gygi S. P., Goldberg A. L. (2013). Why do cellular proteins linked to K63-polyubiquitin chains not associate with proteasomes?. EMBO J..

[cit14] Eddins M. J., Varadan R., Fushman D., Pickart C. M., Wolberger C. (2007). Crystal Structure and Solution NMR Studies of Lys48-linked Tetraubiquitin at Neutral pH. J. Mol. Biol..

[cit15] Datta A. B., Hura G. L., Wolberger C. (2010). The Structure and conformation of Lys-63 linked tetraubiquitin. J. Mol. Biol..

[cit16] Zientara-Rytter K., Subramani S. (2019). The Roles of Ubiquitin-Binding Protein Shuttles in the Degradative Fate of Ubiquitinated Proteins in the Ubiquitin-Proteasome System and Autophagy. Cells.

[cit17] Chen X. (2019). *et al.*, Structure of hRpn10 Bound to UBQLN2 UBL Illustrates Basis for Complementarity between Shuttle Factors and Substrates at the Proteasome. J. Mol. Biol..

[cit18] Dao T. P., Castañeda C. A. (2020). Ubiquitin-Modulated Phase Separation of Shuttle Proteins: Does Condensate Formation Promote Protein Degradation?. BioEssays.

[cit19] Borcherds W., Bremer A., Borgia M. B., Mittag T. (2021). How do intrinsically disordered protein regions encode a driving force for liquid–liquid phase separation?. Curr. Opin. Struct. Biol..

[cit20] Martin E. W. (2020). *et al.*, Valence and patterning of aromatic residues determine the phase behavior of prion-like domains. Science.

[cit21] Ruff K. M., Dar F., Pappu R. V. (2021). Ligand effects on phase separation of multivalent macromolecules. Proc. Natl. Acad. Sci. U. S. A..

[cit22] Dao T. P. (2018). *et al.*, Ubiquitin Modulates Liquid-Liquid Phase Separation of UBQLN2 via Disruption of Multivalent Interactions. Mol. Cell.

[cit23] Krause L. J., Herrera M. G., Winklhofer K. F. (2022). The Role of Ubiquitin in Regulating Stress Granule Dynamics. Front. Physiol..

[cit24] Valentino I. M. (2024). *et al.*, Phase separation of polyubiquitinated proteins in UBQLN2 condensates controls substrate fate. Proc. Natl. Acad. Sci. U. S. A.

[cit25] Dao T. P. (2022). *et al.*, Mechanistic insights into enhancement or inhibition of phase separation by different polyubiquitin chains. EMBO Rep..

[cit26] Habchi J., Tompa P., Longhi S., Uversky V. N. (2014). Introducing protein intrinsic disorder. Chem. Rev..

[cit27] Choi J.-M., Holehouse A. S., Pappu R. V. (2020). Physical Principles Underlying the Complex Biology of Intracellular Phase Transitions. Annu. Rev. Biophys..

[cit28] Leney A. C., Heck A. J. R. (2017). Native Mass Spectrometry: What is in the Name?. J. Am. Soc. Mass Spectrom..

[cit29] Camacho-Zarco A. R. (2022). *et al.*, NMR Provides Unique Insight into the Functional Dynamics and Interactions of Intrinsically Disordered Proteins. Chem. Rev..

[cit30] Naudi-Fabra S., Tengo M., Jensen M. R., Blackledge M., Milles S. (2021). Quantitative Description of Intrinsically Disordered Proteins Using Single-Molecule FRET, NMR, and SAXS. J. Am. Chem. Soc..

[cit31] Robb C. G., Dao T. P., Ujma J., Castañeda C. A., Beveridge R. (2023). Ion Mobility Mass Spectrometry Unveils Global Protein Conformations in Response to Conditions that Promote and Reverse Liquid–Liquid Phase Separation. J. Am. Chem. Soc..

[cit32] Beveridge R. (2014). *et al.*, A mass-spectrometry-based framework to define the extent of disorder in proteins. Anal. Chem..

[cit33] Beveridge R., Calabrese A. N. (2021). Structural Proteomics Methods to Interrogate the Conformations and Dynamics of Intrinsically Disordered Proteins. Front. Chem..

[cit34] Beal R., Deveraux Q., Xia G., Rechsteiner M., Pickart C. (1996). Surface hydrophobic residues of multiubiquitin chains essential for proteolytic targeting. Proc. Natl. Acad. Sci. U. S. A..

[cit35] Castañeda C. A. (2016). *et al.*, Linkage via K27 Bestows Ubiquitin Chains with Unique Properties among Polyubiquitins. Structure.

[cit36] Carvalho A. F. (2011). *et al.*, High-Yield Expression in Escherichia coli and Purification of Mouse Ubiquitin-Activating Enzyme E1. Mol. Biotechnol..

[cit37] Dao T. P. (2018). *et al.*, Ubiquitin Modulates Liquid-Liquid Phase Separation of UBQLN2 via Disruption of Multivalent Interactions. Mol. Cell.

[cit38] Heo C. E. (2021). *et al.*, Ion Mobility Mass Spectrometry Analysis of Oxygen Affinity-Associated Structural Changes in Hemoglobin. J. Am. Soc. Mass Spectrom..

[cit39] El-Baba T. J., Clemmer D. E. (2019). Solution thermochemistry of concanavalin A tetramer conformers measured by variable-temperature ESI-IMS-MS. Int. J. Mass Spectrom..

[cit40] Jung J. E., Ewing M. A., Valentine S. J., Clemmer D. E. (2024). Structural Insights into Linkage-Specific Ubiquitin Chains Using Ion Mobility Mass Spectrometry. J. Am. Soc. Mass Spectrom..

[cit41] Tenno T. (2004). *et al.*, Structural basis for distinct roles of Lys63- and Lys48-linked polyubiquitin chains. Genes Cells.

[cit42] Beveridge R. (2015). *et al.*, Relating gas phase to solution conformations: Lessons from disordered proteins. Proteomics.

[cit43] Pacholarz K. J. (2014). *et al.*, Dynamics of Intact Immunoglobulin G Explored by Drift-Tube Ion-Mobility Mass Spectrometry and Molecular Modeling. Angew. Chem., Int. Ed..

[cit44] Saikusa K. (2018). *et al.*, Structural Diversity of Nucleosomes Characterized by Native Mass Spectrometry. Anal. Chem..

[cit45] Hansen K. (2018). *et al.*, A Mass-Spectrometry-Based Modelling Workflow for Accurate Prediction of IgG Antibody Conformations in the Gas Phase. Angew. Chem., Int. Ed..

[cit46] Konermann L., Ahadi E., Rodriguez A. D., Vahidi S. (2013). Unraveling the Mechanism of Electrospray Ionization. Anal. Chem..

[cit47] Khristenko N. (2023). *et al.*, Native Electrospray Ionization of Multi-Domain Proteins via a Bead Ejection Mechanism. J. Am. Chem. Soc..

[cit48] Fernandez De La Mora J. (2000). Electrospray ionization of large multiply charged species proceeds via Dole's charged residue mechanism. Anal. Chim. Acta.

[cit49] Ghosh D., Rosu F., Gabelica V. (2026). Bead-Ejection Scenario in Electrospray Ionization of Multidomain Nucleic Acids. Anal. Chem..

[cit50] McAllister R. G., Metwally H., Sun Y., Konermann L. (2015). Release of Native-like Gaseous Proteins from Electrospray Droplets via the Charged Residue Mechanism: Insights from Molecular Dynamics Simulations. J. Am. Chem. Soc..

[cit51] Metwally H., Duez Q., Konermann L. (2018). Chain Ejection Model for Electrospray Ionization of Unfolded Proteins: Evidence from Atomistic Simulations and Ion Mobility Spectrometry. Anal. Chem..

[cit52] Zheng T., Galagedera S. K. K., Castañeda C. A. (2021). Previously uncharacterized interactions between the folded and intrinsically disordered domains impart asymmetric effects on UBQLN2 phase separation. Protein Sci..

[cit53] Dao T. P., Rajendran A., Galagedera S. K. K., Haws W., Castañeda C. A. (2024). Short disordered termini and proline-rich domain are major regulators of UBQLN1/2/4 phase separation. Biophys. J..

[cit54] Jarrold M. F. (2022). Applications of Charge Detection Mass Spectrometry in Molecular Biology and Biotechnology. Chem. Rev..

[cit55] Piersimoni L., Kastritis P. L., Arlt C., Sinz A. (2022). Cross-Linking Mass Spectrometry for Investigating Protein Conformations and Protein–Protein Interactions–A Method for All Seasons. Chem. Rev..

[cit56] Mazal H., Haran G. (2019). Single-molecule FRET methods to study the dynamics of proteins at work. Curr. Opin. Biomed. Eng..

