# Supplementary material for: Conformational landscapes resolved by ion mobility mass spectrometry reveal mechanisms of polyubiquitin-controlled phase separation
Source: Chem Sci. 2026 May 19;17(23):11357–67. doi: 10.1039/d6sc00836d (PMC13202630; doi:10.1039/d6sc00836d)
Supplement: SC-017-D6SC00836D-s001 [file SC-017-D6SC00836D-s001.pdf]

Supplementary Information (SI) for Chemical Science.
This journal is © The Royal Society of Chemistry 2026

Conformational landscapes resolved by ion mobility mass spectrometry reveal differential mechanisms of polyubiquitin-controlled phase separation

Christina G. Robb[1], Thuy P. Dao[2], Jakub Ujma[3], Carlos Castañeda[2] and Rebecca Beveridge[1]*

1. Department of Pure and Applied Chemistry, University of Strathclyde, Glasgow G1 1XL, U.K.

2. Departments of Biology and Chemistry, BioInspired Institute, Syracuse University, Syracuse, New York 13244, United States

3. Waters Corporation, Stamford Avenue, Altrincham Road, Wilmslow SK9 4AX, U.K.

*Correspondence: rebecca.beveridge@strath.ac.uk

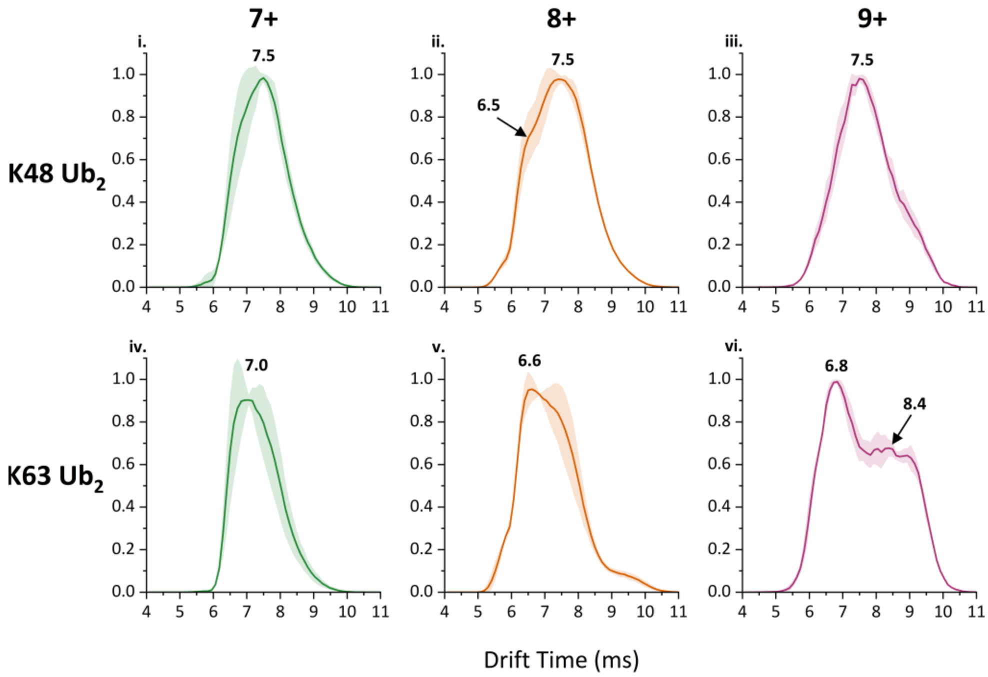

Figure S1: IM-MS of K48- and K63- Ub_2 analysed at an initial concentration of 5 µM from 100 mM AmAc. The number in bold above each peak in is the peak apex. Additional peak features have also been labelled with arrows.

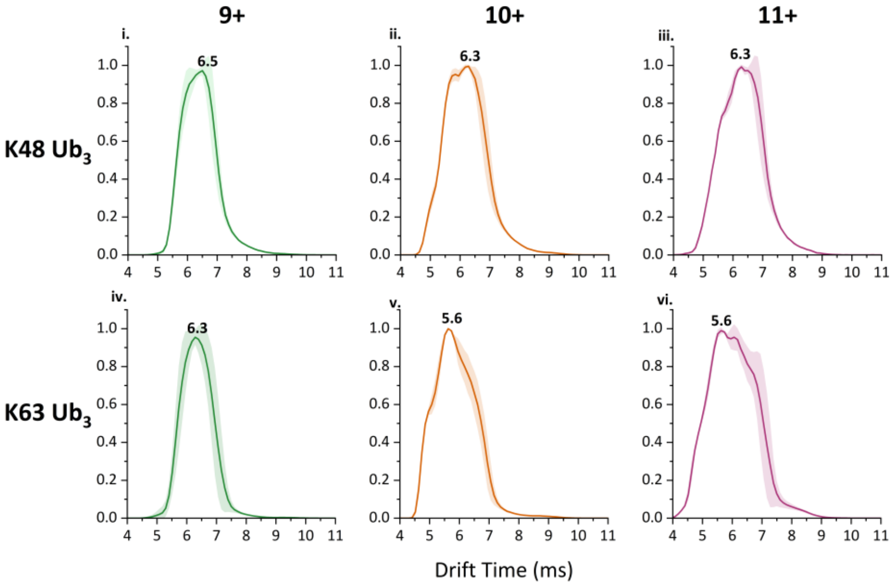

Figure S2: IM-MS of K48- and K63- Ub_3 analysed at an initial concentration of 5 µM from 100 mM AmAc. The number in bold above each peak in is the peak apex.

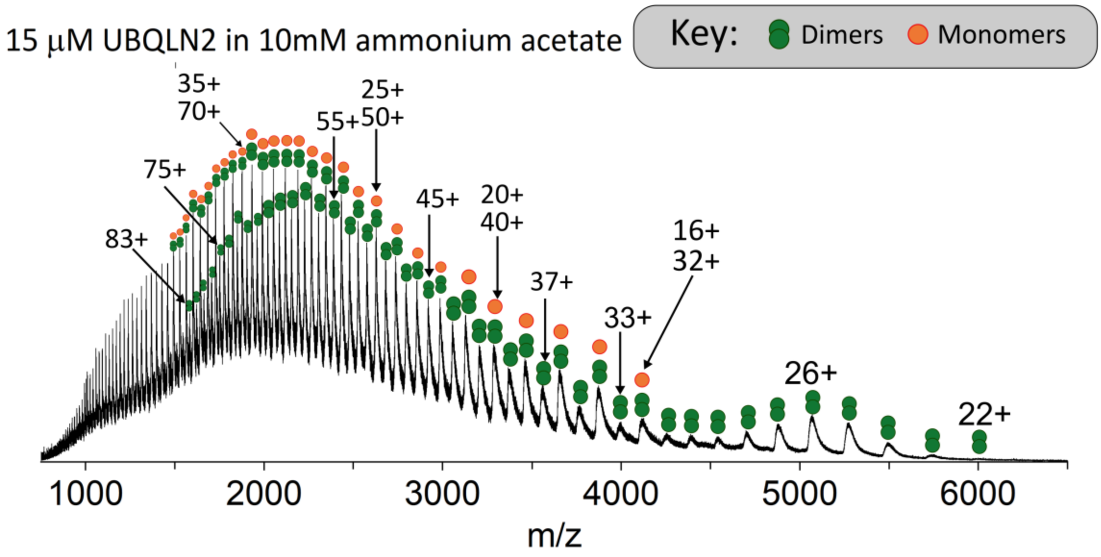

Figure S3: native mass spectrum of UBQLN2 full-length with dimer charge states 22+ to 83+ labelled with double green circles. Coincident monomer charge states are also labelled, with single orange circles. Adapted from reference 32 under license CC-BY 4.0.

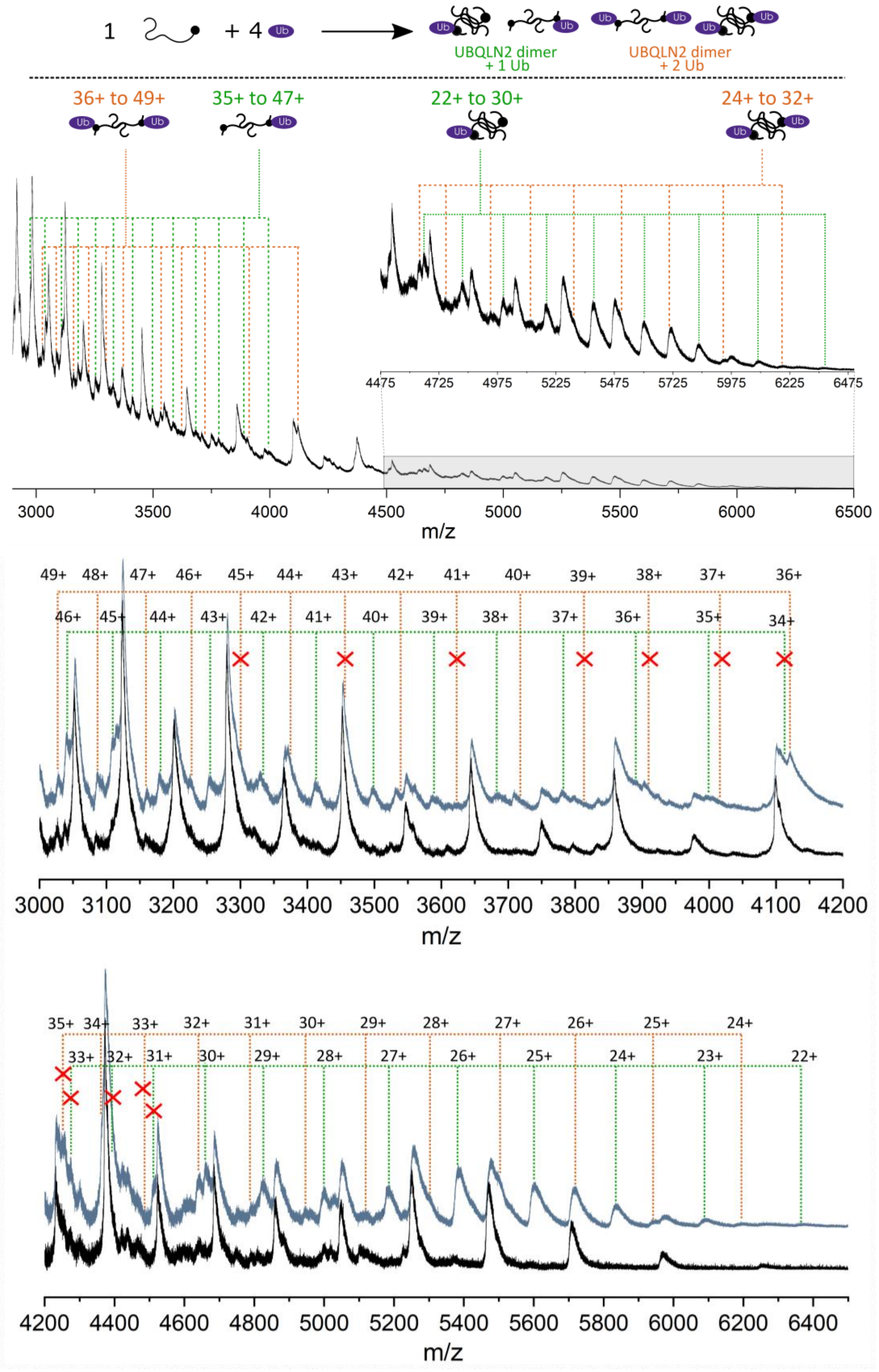

Figure S4: native mass spectra of UBQLN2 and ubiquitin (1:4 molar ratio). Top shows full spectra from 2750-6500 m/z, middle 3000-4200 m/z and bottom 4200-6500 m/z. charge states labelled with red crosses are those which are ambiguous and therefore assigned tentatively. Taken from reference 32 under license CC-BY 4.0.

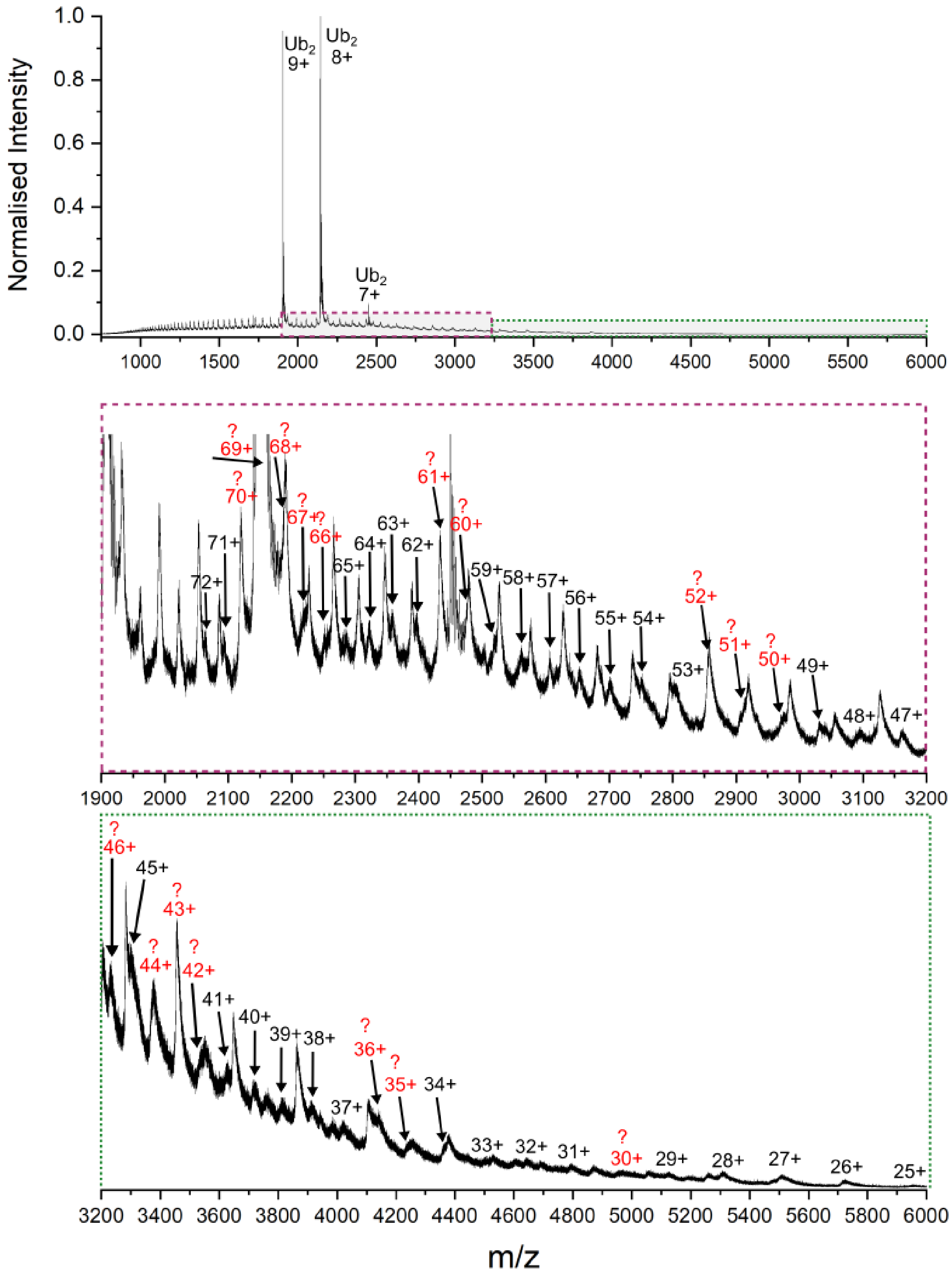

Figure S5: native mass spectra of UBQLN2 and K48-linked diubiquitin (1:1 molar ratio). Top shows full spectra from 750-6000 m/z, middle 1900-3200 m/z and bottom 3200-6000 m/z. Charge states labelled in red are those which are ambiguous and therefore assigned tentatively.

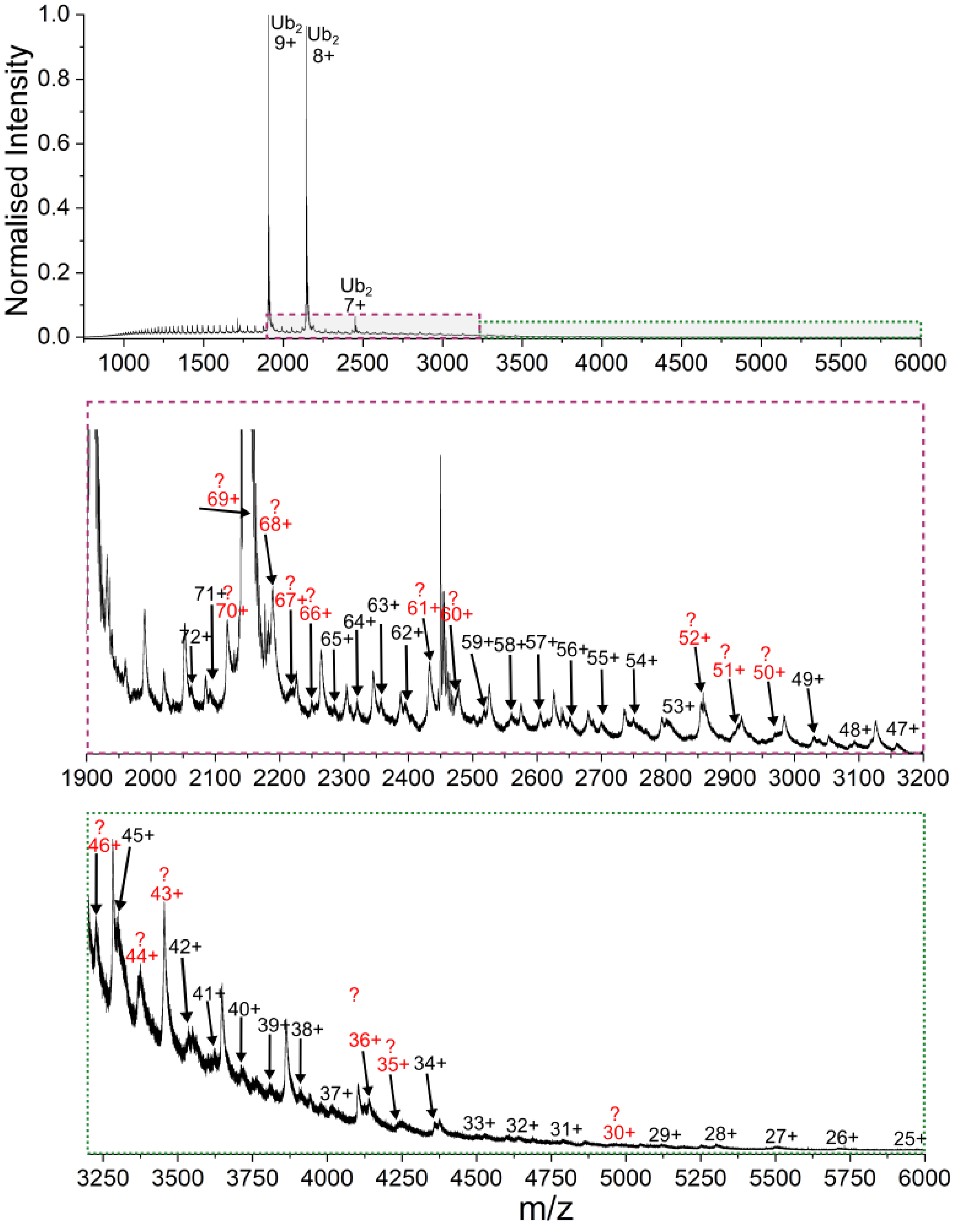

Figure S6: native mass spectra of UBQLN2 and K63-linked diubiquitin (1:1 molar ratio). Top shows full spectra from 750-6000 m/z, middle 1900-3200 m/z and bottom 3200-6000 m/z. Charge states labelled in red are those which are ambiguous and therefore assigned tentatively.

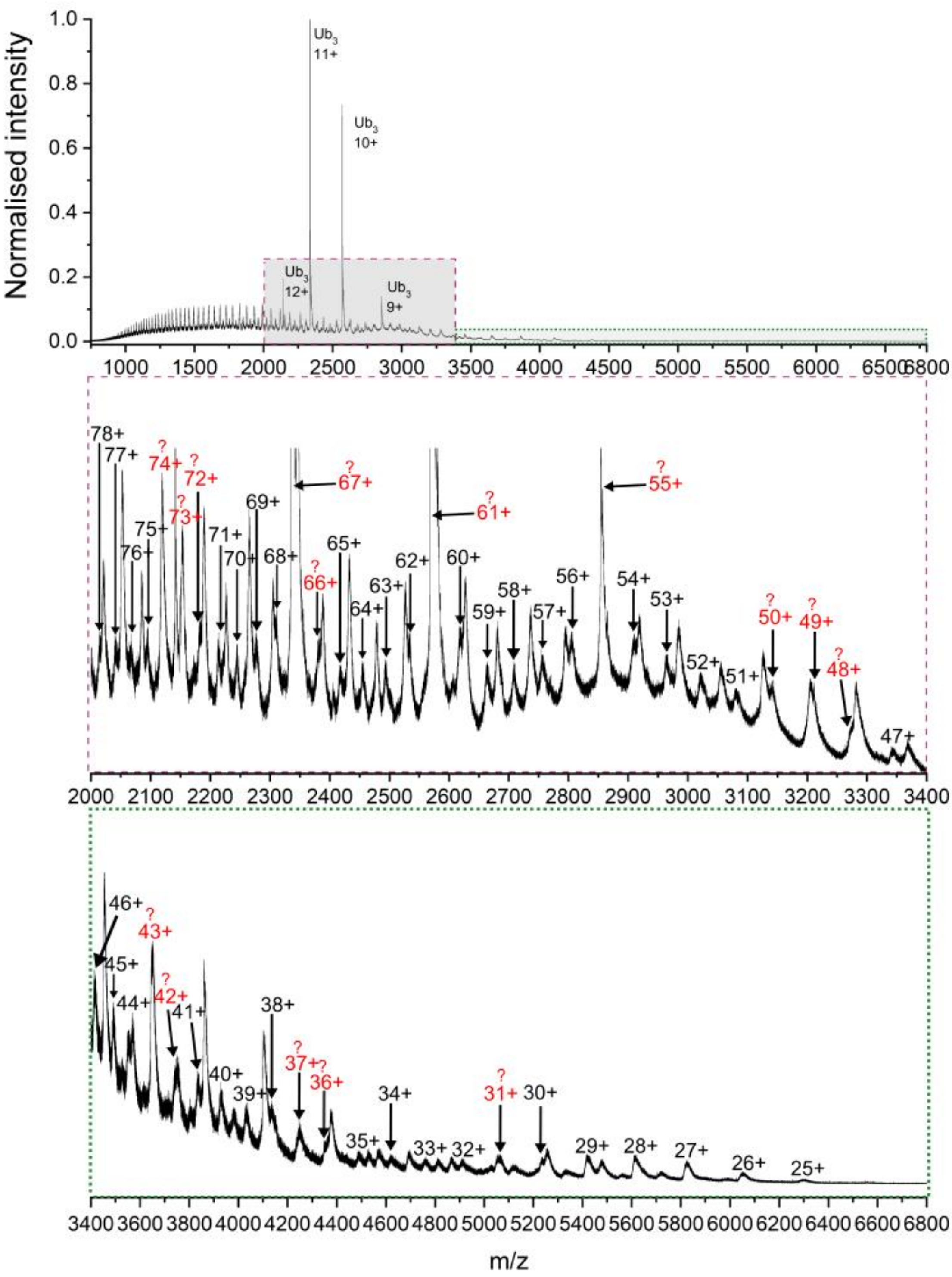

Figure S7: native mass spectra of UBQLN2 and K48-linked triubiquitin (1:1 molar ratio). Top shows full spectra from 750-6000 m/z, middle 2000-3400 m/z and bottom 3400-6800 m/z. Charge states labelled in red are those which are ambiguous and therefore assigned tentatively.

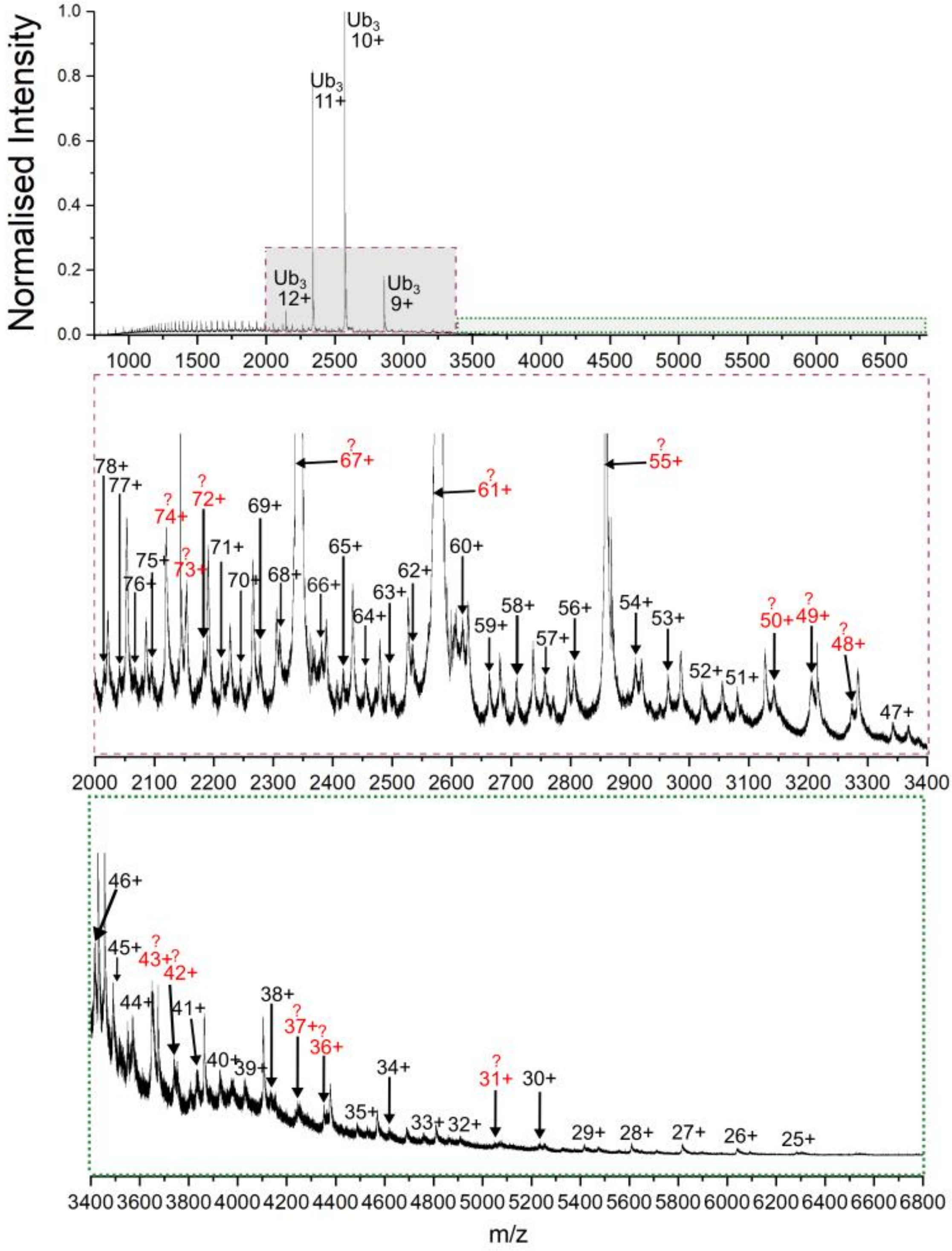

Figure S8: native mass spectra of UBQLN2 and K63-linked triubiquitin (1:1 molar ratio). Top shows full spectra from 750-6000 m/z, middle 2000-3400 m/z and bottom 3400-6800 m/z. Charge states labelled in red are those which are ambiguous and therefore assigned tentatively.

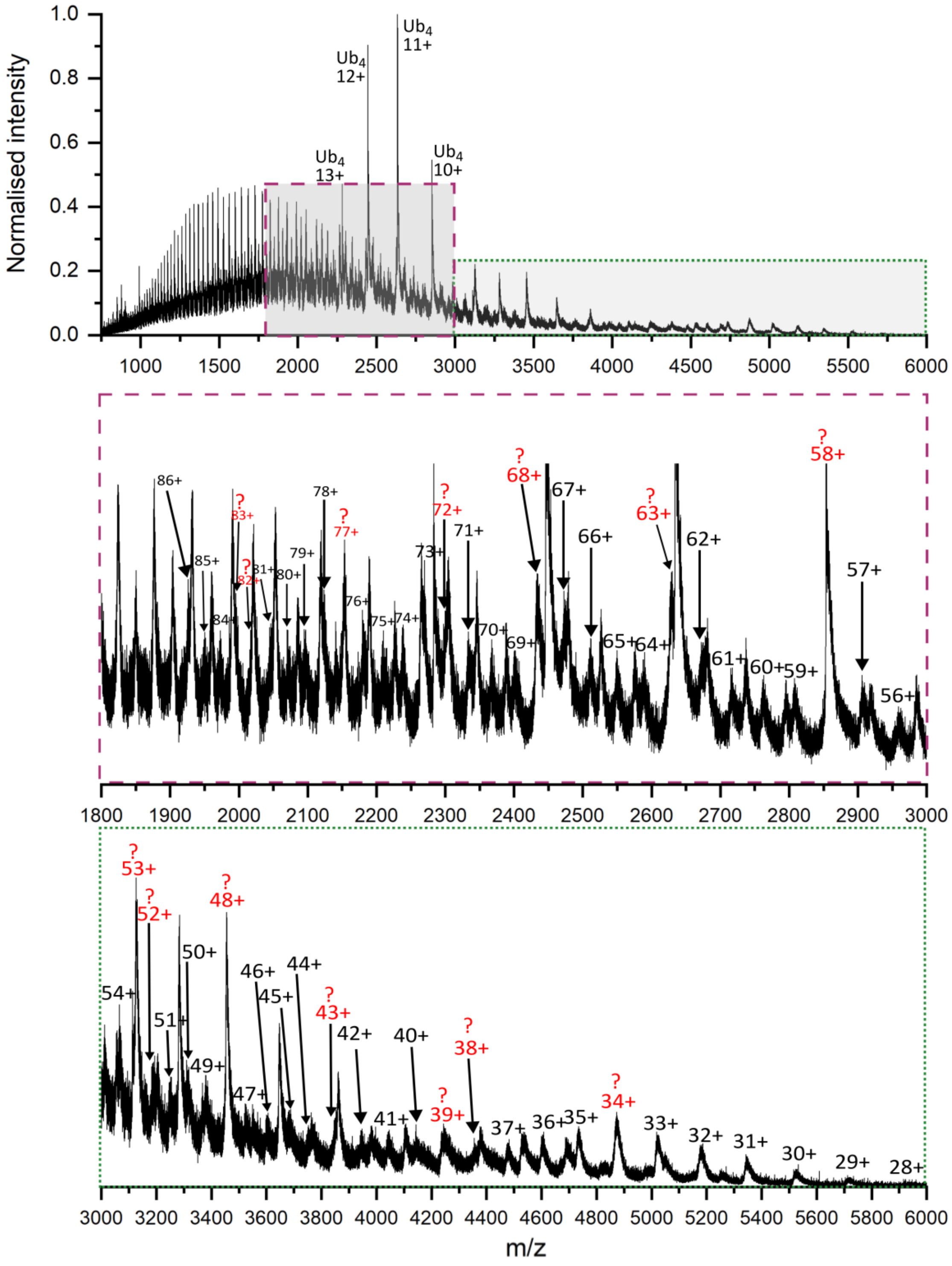

Figure S9: native mass spectra of UBQLN2 and K48-linked tetraubiquitin (1:1 molar ratio). Top shows full spectra from 750-6000 m/z, middle 1900-3200 m/z and bottom 3200-6000 m/z. Charge states labelled in red are those which are ambiguous and therefore assigned tentatively.

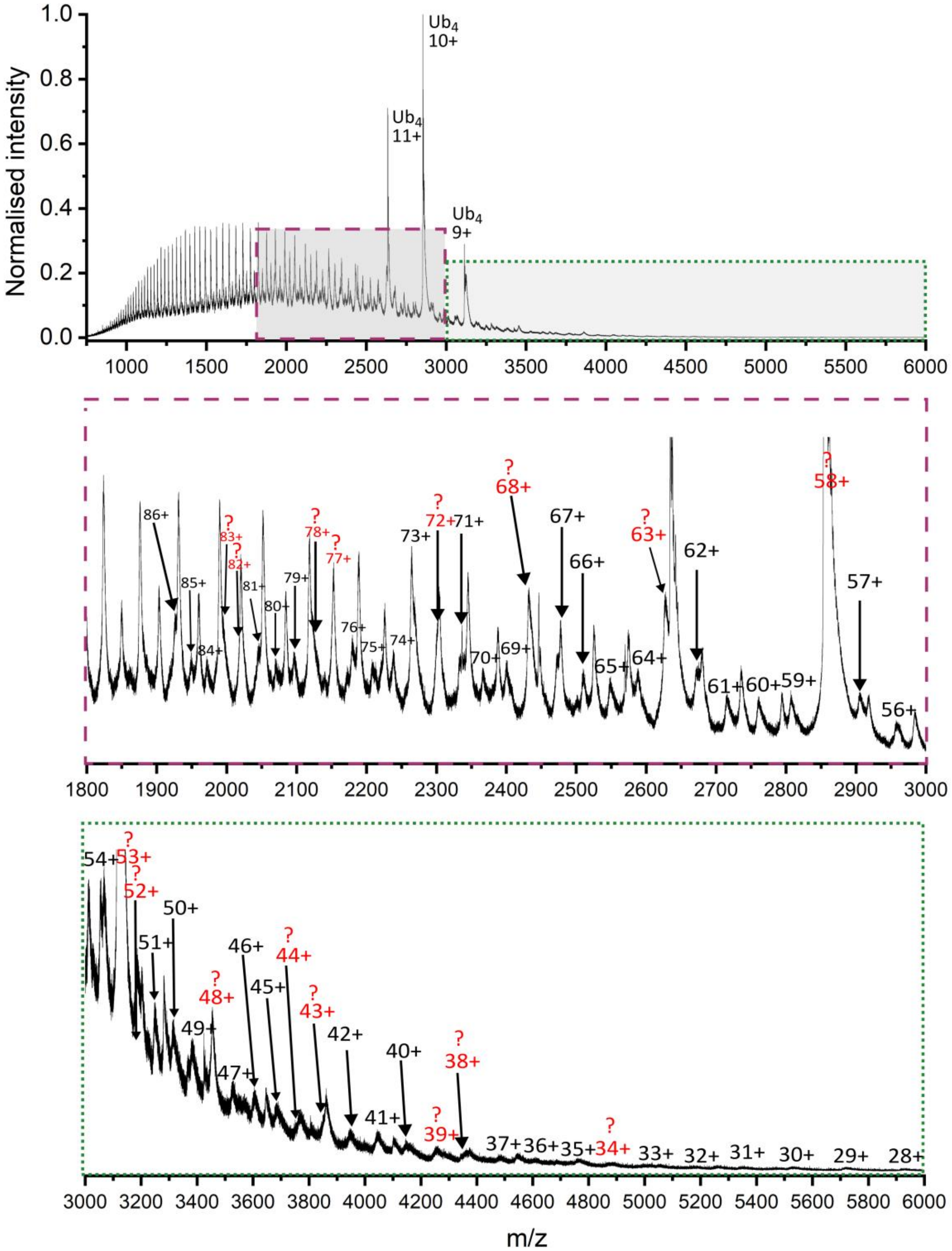

Figure S10: native mass spectra of UBQLN2 and K63-linked tetraubiquitin (1:1 molar ratio). Top shows full spectra from 750-6000 m/z, middle 2000-3400 m/z and bottom 3400-6800 m/z. Charge states labelled in red are those which are ambiguous and therefore assigned tentatively.

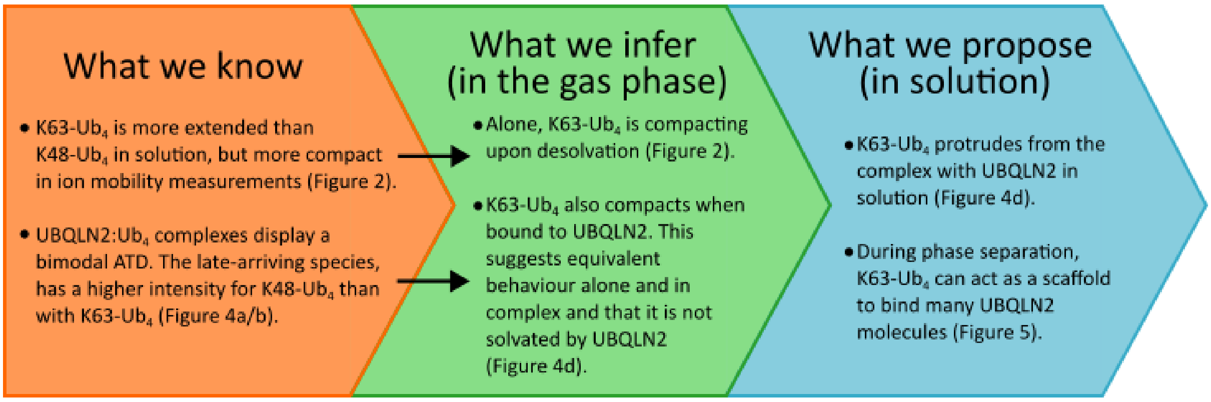

Figure S11: A flowchart to link what we know about polyubiquitin chains alone and in complex with UBQLN2 (orange, left), what we infer is occurring in the gas phase (green, middle) and thus, what we propose is occurring in solution (blue, right).

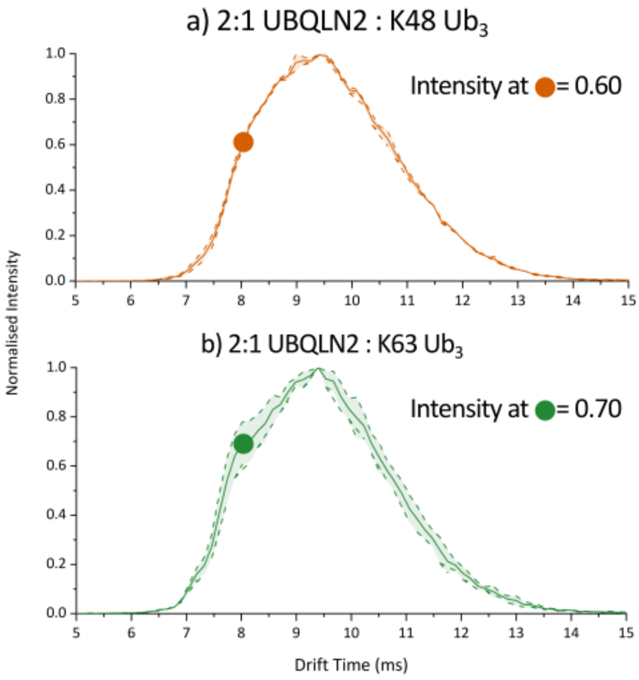

Figure S12: Ion mobility data of 53+ 2:1 a) UBQLN2: K48 Ub_3 complex and b) UBQLN2: K63 Ub_3 complex. a) appears mostly gaussian with no discernible features in the peak shape whereas b) shows a front shoulder to the peak between 7.5 and 9 ms. Normalised intensities at 8 ms (circles) can be compared and show at 0.1 difference in intensity for UBQLN2: K63 Ub_3 complex, which along with the peak shape, indicates a more compact conformation. Solid line represents the average of three measurements, and the shaded area and dotted lines represents the standard deviation across measurements.
As a note, the arrival time for these complexes differs from that of the complexes shown in Figure 4 due to the different charge state (53+ charge state versus 41+ charge state).
